# The Impact of Nanoparticles on Previtreous Behavior: Glass-Forming Nematogenic E7 Mixture-Based Nanocolloids

**DOI:** 10.3390/nano15080597

**Published:** 2025-04-13

**Authors:** Aleksandra Drozd-Rzoska, Joanna Łoś, Sylwester J. Rzoska

**Affiliations:** Institute of High Pressure Physics, Polish Academy of Sciences, Sokołowska 29/37, 01-142 Warsaw, Poland; arzoska@unipress.waw.pl (A.D.-R.); joalos@unipress.waw.pl (J.Ł.)

**Keywords:** glass transition, previtreous behavior, liquid crystals, nematic, nanoparticles, dielectric constant, primary relaxation time, electric conductivity, loss curve maximum, critical phenomena

## Abstract

This report discusses the impact of nanoparticles on glass-forming systems composed of a liquid crystalline (LC) mixture E7 and paraelectric BaTiO_3_ particles (d≈50 nm, globular), tested via broadband dielectric spectroscopy. In the isotropic phase, critical changes in the dielectric constant are shown. They are related to the weakly discontinuous nature of the isotropic–nematic transition. In the nematic phase, two primary relaxation times/processes and DC electric conductivity are considered, down to the glass temperature $T_{g}$. The prevalence of portrayals via the ‘double exponential’ MYEGA equation and the critical & activated Drozd-Rzoska relation for dynamic properties are shown. For the primary loss curve, critical-like changes of its maximum (peak) are evidenced: $\varepsilon_{peak}^{\prime \prime }\propto 1/\left(T-T_{g}^{*}\right)$ for $T_{g}<T<\left(T_{g}+25\;K\right)$, where $T_{g}^{*}<T_{g}$ denotes the extrapolated singular temperature. Dielectric constant monitoring revealed the permanent arrangement of rod-like LC molecules by nanoparticles’ endogenic impact in the nematic phase. The heuristic model regarding this unique behavior is presented. It considers a hypothetical link between the glass transition and a hidden near-critical discontinuous phase transition, uniquely avoiding a symmetry change. The uniaxiality of LC molecules enables the detection of critical-like features when approaching the glass transition, hypothetically associated with a specific ‘amorphous’ phase transition.

## 1. Introduction

At the turn of the 20th and 21st centuries, the glass transition problem was indicated among the grand challenges of 21st-century science [1,2,3]. A cognitive breakthrough was expected in the subsequent decade [1,2,3,4,5,6,7,8,9,10,11,12,13,14,15,16]. The year 2025 has just begun, but understanding glass transition remains puzzling. New experimental results beyond the dominant canon can be essential for the long-awaited cognitive breakthrough. This work presents experimental evidence of the influence of nanoparticles on the previtreous properties of a glass-forming LC system, where such impact can be particularly notable.

The exceptional interest in the glass transition problem is primarily motivated by universalistic features observed in the previtreous domain on approaching the glass temperature ($T_{g}$), for microscopically different systems. One can recall the following: (i) non-Arrhenius changes of primary relaxation time ($\tau ,\;\tau_{\alpha }$), viscosity (*η*), or DC electric conductivity (σ); (ii) decoupling between translational and orientational dynamics; (iii) the non-Debye distribution of the primary relaxation time, (iv) the dynamic crossover, often linked to the “magic” time scale $\tau \left(T_{B}\right)=10^{-7\pm 1}\;s$, (v) the secondary relaxation emerging for $T<T_{B}$, and related to the time scale $\tau_{\beta }\left(T\right)<\tau_{\alpha }\left(T\right)$ [6,7,8,9,11]. Characteristic changes of heat capacity related to the configurational entropy [8,9,17,18], or still puzzling “dynamic heterogeneities” are also noteworthy. The latter is associated with hypothetical multimolecular assemblies, whose direct detection seems limited to physical properties related to experimentally challenging methods coupled to the four-point correlation function, such as nonlinear dielectric spectroscopy (NDS) or its static counterpart—nonlinear dielectric effect (NDE). These properties are associated with changes in dielectric permittivity under a strong electric field [6,19,20,21,22,23,24].

The glass transition occurring on cooling from the deeply supercooled liquid to the amorphous solid is “diffused” in some temperature range, distinguishing the phenomenon from standard continuous or discontinuous phase transitions taking place at precisely defined melting/freezing temperatures. The conventional metric of the glass temperature $T_{g}$ is the center of the ‘diffused domain’ between the deeply supercooled liquid and amorphous solid, detected in heat capacity or density scans at a standard cooling rate. It is empirically correlated with values determined from primary relaxation time (τ) or viscosity (η) scans, namely $\tau \left(T_{g}\right)=100\;s$ and $\eta \left(T_{g}\right)=10^{13}\;Poise$. Notably, in the range $T_{g}<T<T_{B}\approx T_{g}+80\;K,$ changes in the primary relaxation time or viscosity values reach extreme 10 decades [4,5,6,7,8,9,10,11,16,17,18]. Therefore, experimental methods that detect such enormous time scale shifts in single scans are essential to previtreous phenomena studies. It is the generic feature of broadband dielectric spectroscopy (BDS), the most significant method in the given field [6,7,8,9,10,11].

Several models outlining possible glass transition foundations have been formulated [4,5,7,8,9,10,11,12]. However, their experimental validation remains problematic. None of them has addressed quantitatively a set of universalistic properties recalled above. The most common model validation is the general reference to Super-Arrhenius (SA) changes of dynamic properties, primarily by deriving its most popular replacement equation—the Vogel–Fulcher–Tamman (VFT) dependence [7,8,9,10,11,12,25,26,27]:

(1a) $$\tau \left(T\right)=\tau_{\infty }exp\left(\frac{E_{a}\left(T\right)}{RT}\right)\Rightarrow \tau \left(T\right)=\tau_{\infty }exp\left(\frac{D}{T-T_{0}}\right)=\tau_{\infty }exp\left(\frac{D_{T}T_{0}}{T-T_{0}}\right)$$

where the left side is the general SA equation, and the right one is the VFT relation; $E_{a}\left(T\right)$ is the temperature-dependent apparent activation energy; $T>T_{g}$, $T_{0}<T_{g}$ denotes the extrapolated VFT singular temperature; the amplitude $D=D_{T}T_{0}=const$; $D_{T}$ is the fragility strength coefficient, considered the metric of the discrepancy from the basic Arrhenius description related to $E_{a}\left(T\right)=E_{a}=const$.

When comparing the general SA and VFT equations, one obtains the following activation energy approximation:

(1b) $$E_{a}=\frac{RDT}{T-T_{0}}=RDt^{-1}$$

where $t=\left(T-T_{0}\right)/T$ is the metric of the relative temperature distance from $T_{0}$.

Note that parameter $D_{T}$ recalls ‘fragility’, one of the central concepts of *Glass Transition Physics*. It is explained in Appendix A.

Despite the impressive success of the VFT equation in parameterizing experimental data, up to the status of the universalistic symbol of the previtreous dynamics [1,2,3,4,5,6,7,8,9,10,11,12,13,14,15,16,25,26,27], it should be considered only the effective parameterization tool, as explicitly shown in refs. [11,28]. Questioning the informal paradigm of the VFT relation “universality” significantly increased the glass transition cognitive impasse. 

One can expect that inspiration from new experimental results beyond the dominant canon can be essential for approaching breakthrough progress. It can be the case of high-pressure exogenic impact studies, developed mainly via BDS studies for the last decades [11,29,30,31,32,33,34,35,36,37]. A cognitive counterpoint can be studies focused on an endogenic impact factor, such as nanoparticles. However, the experimental evidence remains minimal. The most in-depth studies were probably carried out for relaxation time changes in glycerol + silver nanoparticle colloids [37].

This report discusses the influence of nanoparticles on previtreous properties in glass-forming nanocolloids composed of liquid crystalline (LC) nematogenic mixture E7 and paraelectric BaTiO_3_ nanoparticles. E7 can be supercooled in the nematic phase down to the glass temperature $T_{g}$, at any cooling rate [38,39]. 

In 2000, 135 publications related to LC ‘nematics’ and ‘nanoparticles’ appeared. In subsequent years, the number of research reports increased rapidly, namely [40] in 2010—847 reports, 2020—2560 reports, and 2024—3200 reports. This boosting interest was primarily motivated by expectations that adding nanoparticles to LC compounds can substantially extend and support omnipresent applications of ‘pure’ LC materials [41,42,43]. Nowadays, LC-based nanocolloids are considered a new, specific domain of *Liquid Crystal Physics* and *Materials Engineering* [41,42,43,44,45,46,47,48,49,50,51,52,53,54,55,56,57,58,59,60]. However, the evidence regarding previtreous behavior in LC nanocolloids remains lacking.

This report addresses the mentioned research gaps in general glass transition physics and the new materials area of LC nanocolloids.

## 2. Materials and Methods

E7 is the eutectic mixture is composed of rod-like liquid crystalline (LC) cyanobiphenyl and cyanoterphenol components at a specific composition, namely (1) 4-cyano-4’-n-pentyl-biphenyl (5CB, 51%), (2) 4-cyano-4’-n-heptyl-biphenyl (7CB, 25%), (3) 4-cyano-4’-n-oxyoctyl-biphenyl (8OCB, 16%), and (4) 4-cyano-4’’-n-pentyl-p-terphenyl (5CT, 8%). 

E7 LC mixture was developed for display applications, with an operational range from −5 °C to 50 °C in the nematic phase, supported by convenient dielectric and optical properties. It was first implemented for calculators and watches [61,62,63].

The tested E7 mixture was purchased from Synthon, with the top offered quality. Before measurements, it was degassed and purified in subsequent and repeated steps: (i) solidification by freezing, supported by liquid nitrogen, (ii) removal of air and vapors via a vacuum pump, (iii) heating up to ca. 90 °C, i.e., deeply in the isotropic liquid state.

The tested samples exhibit the following mesomorphism: solid glass—(211.2 K)—nematic (N)—(332.9 K)—isotropic liquid (I), in agreement with values of transition temperatures given in refs. [38,62,63]. Each rod-like LC compound in the E7 mixture is associated with a significant permanent dipole moment (μ≈5 Debye), approximately parallel to the long molecular axis [38,63].

Tested nanocolloids were composed of E7 and BaTiO_3_ nanoparticles (NPs). For nanocolloids, the sedimentation of nanoparticles (NPs) can constitute a problem. One can minimize such a parasitic feature by introducing macromolecular surface agents [41,42,43]. However, it can essentially complicate the registered BDS spectrum. Moreover, even tiny molecular contamination can significantly influence phase transition temperatures [64]. For nanoparticles applied in the given research, avoiding this parasitic factor appears possible for NPs mass fraction concentrations x<1%, following the authors’ experience [65,66,67,68]. For presented studies, mixtures of E7 and nanoparticles, for x<0.5% NPs, were sonicated at a temperature above the isotropic–nematic phase transition for 4 h to obtain homogeneous suspensions. The lack of sedimentation effects and satisfactory distribution of nanoparticles confirm polarized optical microscopy pictures for 5CB, a dominant component of E7 mixture, carried out also under high pressure [67]. For glass-forming systems associated with the presented research, similar images should be detected under very low temperatures, close to $T_{g}$, namely, the extreme range of temperature (down to ~200 K) matched with 12 orders changes in viscosity. This can be a program for further research. On cooling toward $T_{g},$ the viscosity increases up to $\eta \left(T\to T_{g}\right)\to 10^{13}\;Poise$, so the picture shown in ref. [67] cannot be distorted. The stability of nanocolloidal samples was also tested by measuring the dielectric constant and electric conductivity in a special capacitor with rectangular plates: 20 mm in length and 5 mm in width, with two sections. Tests were conducted in the isotropic liquid and the nematic phase to the ‘longitudinal’ and ‘transverse’ positions. For concentrations x≤0.5%, no changes in dielectric properties were noted. A sedimentation-related effect appeared for x→1% after t>1 h. This factor can be removed by acting strong electric field sine–wave packages (U=200 V,   f=1 kHz), lasting at least 10 ms. The results presented in the given report, related to BDS scans, required less than 5 min.

BaTiO_3_ nanopowder (nonconductive, paraelectric, globular, diameter d=50 nm) was purchased from US Research Nanomaterials, Inc. In ref. [69], the structural and phase characterization and the superior size distribution are given. This report discusses nanocolloids with concentrations x≤0.5% of NPs. The specificity of nanocolloid studies yields the possibility of using hundreds of different nanoparticles, in terms of shape, size, properties, and phases [41,42,43,44,45,46,47,48,49,50,51,52,53,54,55,56,57,58,59,60]. The authors chose near-spherical, paraelectric BaTiO_3_ nanoparticles because they minimize the impact of many nanoparticle features on LC surrounding. They also complement the evidence of the authors for the given type of nanocolloids [65,66,67,68].

Broadband dielectric spectroscopy (BDS) is the essential experimental tool for studies on previtreous phenomena due to the enormous time/frequency shift scanning [70]. It is also crucial tool in liquid crystal studies due to its sensitivity to the electric field action [38,39].

In this report BDS Novocontrol spectrometer, enabling the high-resolution complex dielectric permittivity $\varepsilon^{*}=\varepsilon^{\prime }-i\varepsilon^{\prime \prime },$ scans with 5–6 digits resolution was used. Tests were carried out for U=1 V voltage, offering the optimal resolution. Samples were placed in a flat–parallel capacitor made of gold-coated Invar: diameter 2r=20 mm, and the distance between plates d=0.15 mm; i.e., tests were related to bulk samples, for which capacitor plates anchoring ‘parasitic impacts’ are negligible. In LC compounds the biasing of plate-related studies constraints appears for the thin-layer, micrometric, capacitor gaps [38,71]. Notably, ‘micrometric’ gaps are associated with yet another, hardly indicated, constraining factor: the high intensity og the electric field inherently coupled to such condtition.

The intensity of the applied electric field for results presented, $E=6.7\;kVm^{-1}$, is located within the low-intensity limit values. The measurement capacitor was placed within the Quattro Novocontrol temperature control unit and coupled to the BDS spectrometer. It enabled temperature resolution during tests from 0.02 K to 0.05 K, in the temperature range from ~250 °C to −180 °C.

The Novocontrol WinDeta 2016 software controlled the measurement process. The final analysis was carried out using ORIGIN 2025 software.

**Figure 1:**
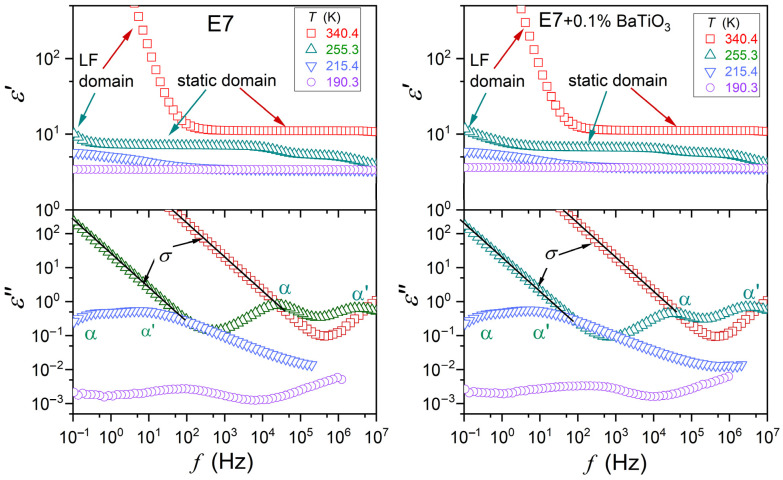
Complex dielectric permittivity spectra, collected in selected temperatures: T=340.3 K for the isotropic liquid phase, T=255.3 K for the nematic phase, T=215.4 K for the supercooled nematic phase, just above the glass transition, and T=190.3K for the solid glass state. Characteristic features are indicated. Note the appearance of two primary relaxation processes (α, α′) and DC electric conductivity (σ).

Figure 1 presents master plots of dielectric spectra—the real ($\varepsilon^{\prime }\left(f\right)$) and imaginary ($\varepsilon^{\prime \prime }\left(f\right)$) parts of dielectric permittivity, as a function of frequency. It illustrates the behavior in subsequent phases for E7 and related nanocolloids. Characteristic features and domains are indicated. For $\varepsilon^{\prime }\left(f\right),$ they are as follows: (i) the static domain, where $\varepsilon^{\prime }\left(f\right)=\varepsilon =const$ in a broad range of frequencies, defining the dielectric constant ε, and (ii) the low frequency (LF) domain with the boost of $\varepsilon^{\prime }\left(f\right)$ values. The emerging contribution of ionic contamination is most often cited as an explanation of the latter [38,39,48,70], but the authors would like to indicate that translational shifts of molecules from their equilibrium positions can yield a similar contribution.

For the imaginary part of dielectric permittivity, primary loss curves enable the direct insight into the dynamics of an average molecule responding to the external electric field. Peaks (maxima) of loss curves determine the primary ($alpha,\;\alpha_{\tau }$) relaxation time characterizing the ability to the orientation process: $\tau =1/\omega_{peak}$, ω=2πf. Branches of the loss curves reflect the distribution of these relaxation times. Jonsher indicated the following scaling of loss curves [66,72,73]:

(2) $$\varepsilon^{\prime \prime }\left(f<f_{peak}\right)\propto f^{m}\;\;\;\Rightarrow \;\;\;{log}_{10}\varepsilon^{\prime \prime }\left(f\right)=c+m{log}_{10}f,$$

(3) $$\varepsilon^{\prime \prime }\left(f>f_{peak}\right)\propto f^{-n}\;\;\;\Rightarrow \;\;\;{log}_{10}\varepsilon^{\prime \prime }\left(f\right)=c^{\prime }-n{log}_{10}f,$$

where $c,c^{\prime }=const$ and m,n≤1 are coefficients related to the distribution of relaxation times. For a single relaxation time Debye process m,n=1.

The above dependencies enable estimating the peak frequency and then the relaxation time using the derivative of experimental data via the following relation [11]:

(4) $$d{log}_{10}\varepsilon^{\prime \prime }\left(f\right)/d{log}_{10}f=0\;\mathrm{for}\;f=f_{peak}$$

Relations (2)–(4) offer a protocol for determining the primary relaxation times, avoiding the uncertainty associated with the nonlinear fitting. The alternative path is associated with portraying loss curves via the Havriliak–Negami (HN) equation [11,66,74,75]:

(5) $$\varepsilon^{*}\left(\omega \right)=\varepsilon_{\infty }+\frac{\Delta \varepsilon }{{\left[1+{\left(i\omega \tau \right)}^{a}\right]}^{b}}+\frac{\sigma_{DC}}{i\varepsilon_{0}\omega^{\phi }},$$

where ω=2πf, $\varepsilon_{\infty }$ is the value of permittivity at the high-frequency limit; it is related to the sum of atomic and electronic polarizabilities. ϕ is related to the distortion from hypothetical ionic translational impacts; the DC conductivity limit is related to ϕ=1; parameters a,b≤1 describe the distribution of relaxation time; a,b=1 are for the single relaxation time Debye model.

Jonsher and HN distribution parameters are linked: m=b and n=ab [11,66]. Relaxation time determined via HN equation requires multi-parameter nonlinear fitting, associated with a notable error. Nevertheless, it is the only tool when loss curves overlap, limiting the reliable application of the approach based on Equations (2)–(4). 

The transformation $\varepsilon^{\prime \prime }\left(f\right)\;\to \sigma^{\prime }\left(f\right)=\omega \varepsilon^{\prime \prime }\left(f\right)$ enables determining the DC electric conductivity, manifesting as the horizontal line in the plot ${log}_{10}\sigma^{\prime }\left(f\right)$ vs. ${log}_{10}f$. In the DC domain: $\sigma^{\prime }\left(f\right)=\sigma_{DC}=\sigma =const$. 

## 3. Results and Discussion

### 3.1. Dielectric Constant Changes

Dielectric constant ε is related to the real part of dielectric permittivity $\varepsilon^{\prime }\left(f\right)$ in the static frequency domain, where $\varepsilon^{\prime }\left(f\right)=\varepsilon \approx const$. Figure 1 shows that for systems tested in the given report, it occurs for frequencies between ~1 kHz and ~1 MHz, in the isotropic liquid phase. For dipolar liquids, $\varepsilon \left(T\right)$ pattern of changes can indicate the preferred arrangement of permanent dipolar moments coupled to molecules; namely, (*i*) ‘antiparallel’ for $d\varepsilon \left(T\right)/dT>0$, and (*ii*) ‘parallel’ for $d\varepsilon \left(T\right)/dT<0$; i.e., where they mostly follow the external electric field [76].

**Figure 2:**
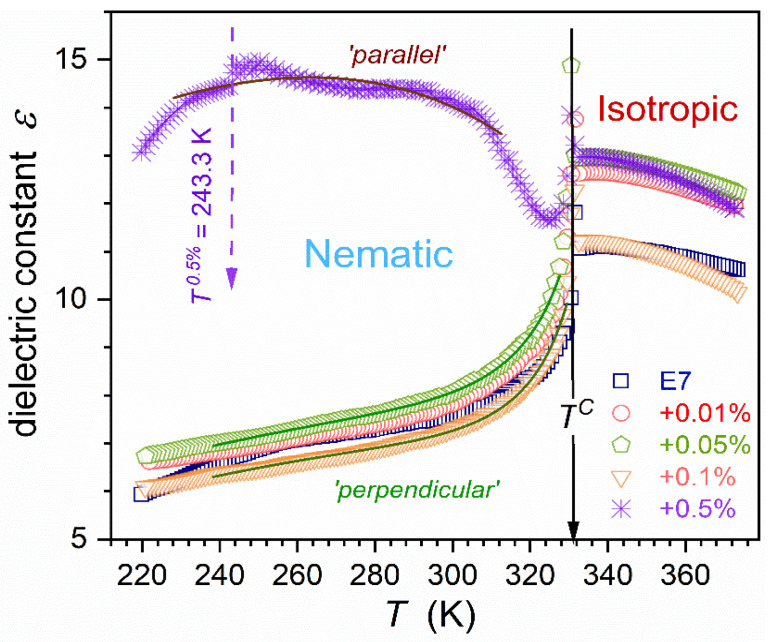
Dielectric constant changes in the isotropic liquid and nematic phases of E7 and related nanocolloids with BaTiO_3_ nanoparticles. Concentrations are given in the plot. The solid arrow indicates the clearing temperature at $T^{C}=332.9\;K$, related to weakly discontinuous I-N phase transition. The dashed arrow indicates a discontinuous change in the dielectric constant for x=0.5% nanocolloid. Curves portraying (brown (up), green (down)) data are related to Equations (11) and (12). In the figure, ‘*Parallel*’ means the behavior resembling dielectric constant changes in the nematic phase observed for samples oriented by the strong external magnetic field, in such a way that the scanning electric field and the long molecular axis are parallel. The name ‘*Perpendicular*’ refers to the behavior occurring when the scanning electric field and the long molecular axis are perpendicular [38,39].

Figure 2 presents changes in the dielectric constant for E7 and tested nanocolloids, from the isotropic liquid phase down to the glass temperature in the supercooled nematic phase. In the isotropic liquid phase, for each concentration of nanoparticles (NPs), the same pattern of pretransitional changes occurs [77,78,79]:

(6) $$\varepsilon \left(T\right)=\varepsilon^{*}+a\left(T-T^{*}\right)+A{\left(T-T^{*}\right)}^{\varphi },$$

where $T^{*}<T^{C}$ is the extrapolated continuous phase transition temperature, $T^{C}$ is the clearing temperature linked to the weakly discontinuous phase transition in the given case $T^{C}=T_{I-N}$; parameters a,A=const; the exponent φ=1−α, where α=1/2 is the heat capacity critical exponent. The value $\Delta T=T^{C}-T^{*}$ is the metric of the isotropic–nematic (I-N) transition discontinuity, a significant reference for theoretical models [38,39].

Such behavior was noted for rod-like LC compounds with a permanent dipole moment approximately parallel to the long molecular axis, like 5CB or 8OCB included in the E7 mixture [77,78,79]. In the isotropic liquid phase, the pretransitional/precritical growth of prenematic fluctuations is associated with the weakly discontinuous nature of I-N transition [38,39]. It leads to the following rise of prenematic fluctuations volume:

(7) $$\xi \xi \left(T\right)=\xi_{0}{\left(T-T^{*}\right)}^{-\nu =-1/2}\Rightarrow V\left(T\right)\propto {\left[\xi \left(T\right)\right]}^{3}\propto {\left(T-T^{*}\right)}^{-3\nu =-3/2},$$

where ξ is the correlation length of pretransitional fluctuations related to the critical exponent ν, with the mean-field value (in the given case); $V\left(T\right)$ approximates the volume of prenematic fluctuations.

Within prenematic fluctuations, the antiparallel arrangement of permanent dipole moments [38,39] leads to the ‘cancellation’ of permanent dipole moments contribution to dielectric constant [79]. Consequently, the dielectric constant within fluctuations is significantly smaller than for the isotropic liquid surrounding. One can consider the *Contrast Factor* (CF) as the metric for the difference between the physical characterization of pretransitional fluctuations and their (isotropic liquid) surrounding. In the given case, it can be related to the dielectric constant,. This is the case for all LC compounds related to the E7 mixture and the behavior described by Equation (6), possible when CF≠0. 

For rod-like nematogenic compounds with the transverse permanent dipole moment, in respect to the long molecular axis, the mentioned cancellation does not occur. Consequently, CF=0 and the pretransitional anomaly of dielectric constant in the isotropic phase of such LC materials is absent [68].

**Figure 3:**
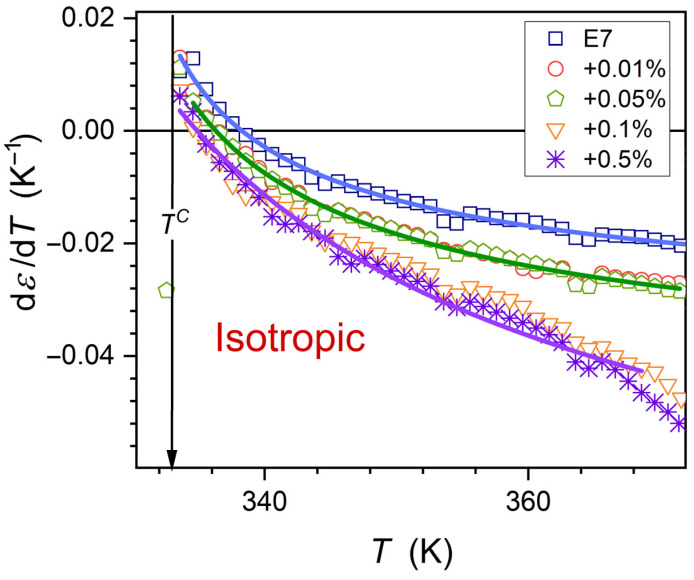
The derivative of dielectric constant changes in the isotropic phase of E7 and its nanocolloids with BaTiO_3_ nanoparticles. Curves portraying experimental data are related to Equation (8), with parameters given in Table 1.

The volume occupied by prenematic fluctuations rapidly grows on approaching phase transition $T\to T^{*}$, as shown in Equation (7). In the E7 LC mixture, ‘prenematic’, fluctuations-related contribution to the registered value of the dielectric constant can dominate close to the clearing temperature. It is related to the following crossover: $d\varepsilon \left(T\right)/dT>0\leftarrow d\varepsilon \left(T\right)/dT<0$. Such behavior and the portrayal by Equation (6) are validated in Figure 3, where the results the derivative analysis is also shown [79]:

(8) $$d\varepsilon \left(T\right)/dT=a+B{\left(T-T^{*}\right)}^{\varphi -1}\propto {\left(T-T^{*}\right)}^{-1/2},$$

where $B=A\varphi =A\left(1-\alpha \right)$ and the exponent φ−1=−α.

Related parameters are given in Table 1. The lack of impact of nanoparticles on values of the clearing temperature ($T^{C},\;T_{I-N}$) and exponent α is notable. The same behavior was observed in earlier studies in other LC compounds (5CB and 8OCB)-based nanocolloids, for which discussed here supercooling is absent [65,66,67]. A notable influence of nanoparticles on the phase transition discontinuity metric is visible: $\Delta T^{*}=T^{C}-T^{*}$, from $\Delta T^{*}\left(x=0\%\right)\approx 3.4\;K$ to $T^{*}\left(x=0.5\%\right)\approx 17\;K$.

Note that Figure 3 is for the numerical derivative of experimental data presented in Figure 3, focusing on the isotropic liquid phase. Hence, the slight discrepancies between transformed data and the proposed scaling function Equation (8) are the consequence of such analysis, not a specific phenomenon behind it.

It is worth recalling that decades of discussions about the nature of the isotropic–nematic (I-N) phase transition have indicated two types of descriptions: ‘mean-field’ (MF) and ‘trictitical’ (TCP). There is a ‘’nomenclature’ problem here, which may be confusing for some readers. The decisive criterion for the mean-field behavior is the Ginzburg criterion ([64,80,81] and refs therein). It compare the sizes of pretransitional/critical fluctuations and dominant intermolecular interactions. In such a context, both the aforementioned descriptions, named ‘MF’ and ‘TCP’, are of the general mean-field nature. The standard ‘MF’ behavior is associated with a single critical point, whereas TCP is the result of three continuous phase transition lines intersection. Hence, the notation of mean-field types 1 and 2 (MF1 and MF2_might be more optimal. However, the ‘historical’ nomenclature mentioned above is used. Differences between MF (MF1) and TCP (MF2) behavior are manifested in values of exponents describing pretransitional changes:

- MF description: isotropic phase: γ=1 (compressibility), α=0 (heat capacity/internal energy), and ν=1/2 (correlation length), and for the nematic phase: β=1/2 (order parameter), α=1/2, and ν=1/2 (correlation length). The value α=1/2 in the isotropic phase, consistent with the experimental evidence, appears only after introducing the ‘fluctuational corrections’ [80,81,82].
- TCP description: γ=1, α=1/2 (and ν=1/2; for the nematic phase: β=1/4, α=1/2, and ν=1/2 [80,81,82].

In the nematic phase of rod-like LC materials, the dielectric constant can be tested using two general research paths: (i) in non-oriented samples, which is associated with the spontaneous arrangement of molecules and coupled dipole moments, and (ii) in oriented samples with respect to the long molecular axis of rod-like LC molecules. The latter can be realized in the nematic phase via the strong magnetic field (a few Tesla) or by covering the capacitor plates with a polymeric layer interacting with LC molecules to yield the desired orientation [38,39]. The latter is dedicated to thin-layer (micrometric) samples. [38,83,84]. As noted in the Methods section, such constraints can influence observed patterns in the nematic phase, which are absent when a bulk sample is ‘oriented’ by a strong magnetic field. For the latter, one can consider the model behavior in frames of the Landau–de Gennes model [38,39], focusing on the order parameter $\Delta \varepsilon \left(T\right)$ and the ‘diameter’ $\delta \left(T\right)$ [82]:

(9) $$\Delta \varepsilon \left(T\right)=\varepsilon_{⫽}-\varepsilon_{⊥}\approx \varepsilon_{op}^{**}+B{\left(T^{**}-T\right)}^{\beta },$$

(10) $$\delta \left(T\right)=\frac{1}{3}\varepsilon_{⫽}+\frac{2}{3}\varepsilon_{⊥}\approx \varepsilon_{d}^{**}+D{\left(T^{**}-T\right)}^{1-\alpha }+d\left(T^{**}-T\right),$$

where $T<T^{C}$, $T^{C}$ is the I-N discontinuous transition ‘clearing’ temperature, $\Delta \varepsilon \left(T\right)$ is the order parameter (*op*) metric, and $\delta \left(T\right)$ denotes the ‘diameter’, B,d,D are constant amplitudes; $T^{**}$ is the hypothetical continuous transition extrapolated from the nematic phase.

The component $\varepsilon_{⫽}$ is related to LC samples in the nematic phase, oriented by the strong magnetic field so that the long axis of rod-like molecule and the probing electric field impact in the measurement capacitor are *parallel* [38,39]. The component $\varepsilon_{⊥}$ is related to LC samples in the nematic phase oriented by the strong magnetic field so that the long axis of rod-like molecule and the scanning of the electric field in the measurement capacitor are *perpendicular*; i.e., properties related to the short molecular axis can be tested [38,39]. High resolution and distortions-sensitive analysis carried out for 5CB and 8OCB yielded the order parameter exponent β=1/4 and heat capacity exponent α=1/2, which indicates tricritical point (TCP) mean-field-type approximation ([81] and refs. therein).

Linking Equations (9) and (10), one obtains relations for modeling the ‘parallel’ and ‘perpendicular’ components evolution in the nematic phase:

(11) $$\varepsilon_{⊥}\approx a_{1}-a_{2}{\left(T^{**}-T\right)}^{\beta }+a_{3}{\left(T^{**}-T\right)}^{1-\alpha }+a_{4}\left(T^{**}-T\right),$$

(12) $$\varepsilon_{⫽}=b_{1}+b_{2}{\left(T^{**}-T\right)}^{\beta }+b_{3}{\left(T^{**}-T\right)}^{1-\alpha }+b_{4}\left(T^{**}-T\right),$$

where $a_{i}$ and $b_{i}$ are empirical parameters related to Equations (9) and (10).

For E7 and its nanocolloids up to x=0.1% NPs, the fair portrayal via Equation (11) takes place, as shown in Figure 2. Notable that for ‘pure’ E7 (x=0%), the decrease of the capacitor gap distorts such a pattern, increasing detected values. For the concentration x=0.5% of NPs, dielectric constant ‘roughly’ follows the ‘parallel’ pattern (Equation (12)), with distortions near I-N transition and a ‘slight’ discontinuous transition at T=243.3 K. It is confirmed by the DSC test, shown in Appendix A.

The ability of BaTiO_3_ nanoparticles for the permanent endogenic orientation of LC samples was earlier reported by the authors for 8OCB and 5CB based nanocolloids [65,66,67,68].

### 3.2. Primary Relaxation Time in the Previtreous Domain

**Figure 4:**
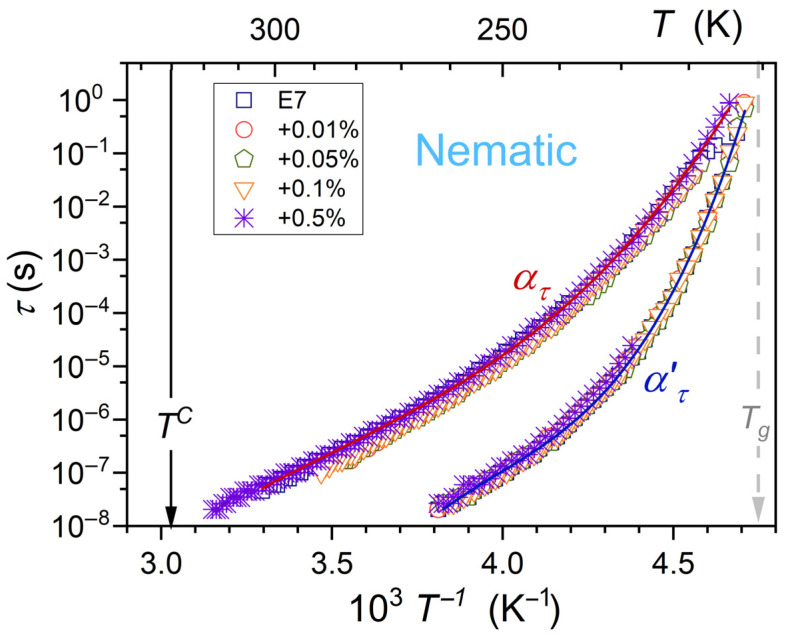
The primary relaxation times change for *alpha* ($\alpha_{\tau }$) and *alpha’* ($\alpha_{\tau }^{\prime }$) processes in the nematic phase of E7 and related nanocolloids with BaTiO_3_ nanoparticles. $T_{g}$ is for the glass temperature and $T^{C}=T_{I-N}$ denotes the clearing temperature related to the I-N weakly discontinuous phase transition. The nonlinear behavior in the given scale indicates the super/non-Arrhenius pattern. Curves portraying data are related to Equation (13a).

Dielectric constant is the static (frequency-independent) property associated with the static domain of the real part of dielectric permittivity (see Figure 1). Dynamic properties explicitly manifest for the imaginary part of dielectric permittivity, as shown in Figure 1. The primary loss curve peak reflects the relaxation time of permanent dipole moments, coupled to rod-like LC molecules, under the impact of the electric field. For E7 and related nanocolloids, there are two loss curves (*alpha* ($\alpha_{\tau }$) and *alpha’* ($\alpha_{\tau }^{\prime }$)) in the nematic phase, as noted in Figure 1. It can be associated with the multicomponent nature of E7 mixture. The temperature behavior of primary relaxation times related to $\alpha_{\tau }$ and $\alpha_{\tau }^{\prime }$ are presented in Figure 4. It is explicitly non-Arrhenius, as shown by the nonlinear behavior related to the so-called Arrhenius scale, related to Equation (1) and applied in Figure 4. The overlapping of relaxation time dependencies is notable; i.e., they are almost non-impacted by nanoparticle concentrations.

In ref. [11], the distortions-sensitive analysis of various model equations portraying previtreous dynamics in glass-forming liquids was carried out. It showed the limited validity of the VFT portrayal (Equation (1)) and indicated the universalistic prevalence of the double-exponential MYEGA equation [85,86]:

(13a) $$\tau \left(T\right)=\tau_{\infty }exp\left(\frac{K^{\prime }}{T}exp\left(\frac{C}{T}\right)\right),$$

The fair parameterizations via this relation are shown in Figure 4. They are associated with the following parameters $\tau_{\infty }=0.34\;ns,\;\;K^{\prime }=120\;K,\;\;C=780\;K$ for $\alpha_{\tau }$ relaxation, and $\tau_{\infty }=3.8\;ns,\;\;K^{\prime }=0.1\;K,\;\;C=2250\;K$ for $\alpha_{\tau }^{\prime }$ primary relaxation processes.

The comparison of the above relation and the general SA dependence (the left side of Equation (1)) yields the apparent activation energy for the MYEGA equation:

(13b) $$E_{a}\left(T\right)=RKexp\left(\frac{C}{T}\right)=\frac{RK^{\prime }}{exp\left(-C/T\right)}\Rightarrow E_{a}\left(T\right)\approx \frac{RK^{\prime }}{1+C/T+\ldots }\approx \frac{RK^{\prime }}{\left(T-C\right)/T}=RK^{\prime }t^{-1}.$$

The right side of Equation (13b) indicates the transformation MYEGA→VFT, leading to the apparent activation energy given in Equation (1b). Notable, that the VFT equation emerges when limited to the first-term Taylor expansion of MYEGA-related apparent activation energy, as shown above. The comparison of Equations (1) and (13b) shows the link between VFT and MYEGA dependencies, indicating the following relationship between relevant parameters: $C=T_{0}$ and $K^{\prime }=D=D_{T}T_{0}$. It is worth stressing that MYEGA equation is not associated with a finite temperature singularity, which is the inherent feature of the VFT behavior. As shown above, it appears due to the ‘nonlinear’ terms truncation’ in the Taylor expansion.

### 3.3. Primary Loss Curve Maximum Previtreous Changes

**Figure 5:**
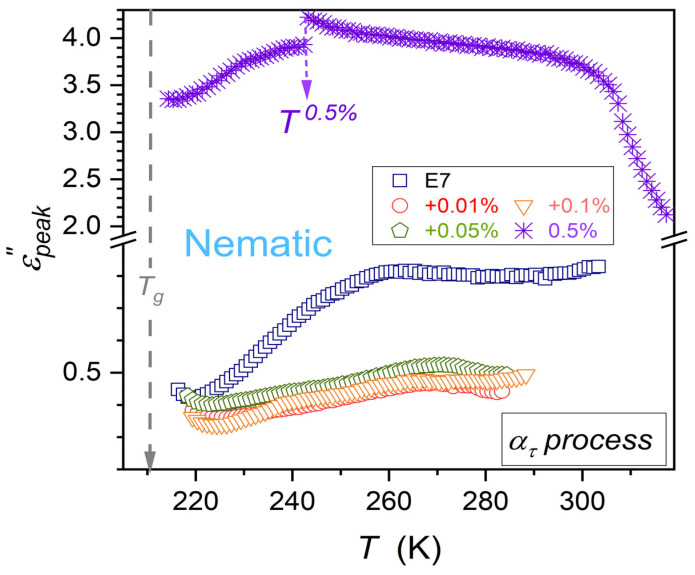
Evolutions of $\alpha_{\tau }$ process primary loss curves maximum in the nematic phase of E7 and related nanocolloids with BaTiO_3_ nanoparticles. The discontinuous change emerging for x=0.5% (mass fraction) concentration is indicated.

**Figure 6:**
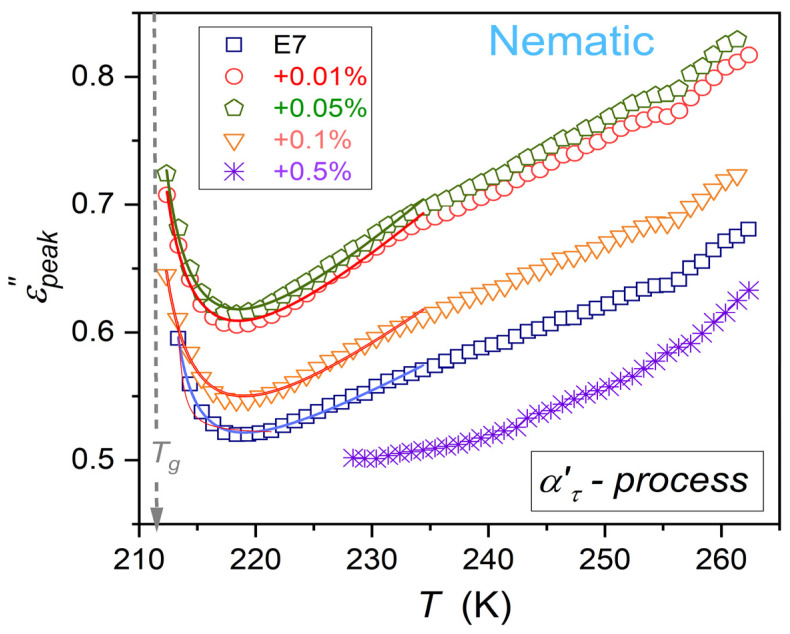
Evolutions of $\alpha_{\tau }^{\prime }$ process primary loss curve maxima in the nematic phase of E7 and related nanocolloids with BaTiO_3_ nanoparticles in the nematic phase down to the glass temperature. Curves portraying data near $T_{g}$ are related to Equation (14), with parameters given in Table 2.

Coordinates of the loss curve maximum $\left(\tau ,\;\;\varepsilon_{peak}^{\prime \prime }\right)$ define the primary relaxation time (τ) discussed above and the metric of the energy related to this process ($\varepsilon_{peak}^{\prime \prime }$) [66,67,68]. Temperature changes of the primary relaxation time $\tau \left(T\right)$ are almost independent from NPs concentration, as visible in Figure 4. The loss curve peak $\varepsilon_{peak}^{\prime \prime }\left(T\right)$ patterns of changes strongly depend on NPs concentration. It is shown in Figure 5 and Figure 6, for $\alpha_{\tau }$ and $\alpha_{\tau }^{\prime }$ relaxation processes.

For $\alpha_{\tau }$ process, the addition of nanoparticles first decreases the values of $\varepsilon_{peak}^{\prime \prime }\left(T\right)$, but for the highest tested concentration, x=0.5%, it strongly rises, as presented in Figure 5. Moreover, the discontinuous transition at $T^{0.5\%}=243.3\;K$ appears both for $\varepsilon \left(T\right)$ (Figure 4) and $\varepsilon_{peak}^{\prime \prime }\left(T\right)$ (Figure 5).

Patterns of $\varepsilon_{peak}^{\prime \prime }\left(T\right)$ changes for $\alpha_{\tau }$ and $\alpha_{\tau }^{\prime }$ are notably different. However, for both primary processes, the previtreous ‘bending up’ when approaching $T_{g}$ appears. It can suggest a significant change in the energy required for electric field-related reorientation.

The reliable estimations of $\varepsilon_{peak}^{\prime \prime }\left(T\right)$ in the immediate vicinity of $T_{g}$ were possible only for $\alpha_{\tau }^{\prime }$ processes, due to its explicit manifestation (Figure 1). As shown by solid curves portraying experimental data in Figure 6, the previtreous ‘anomaly’ can be parameterized by the following relation:

(14) $$\varepsilon_{peak}^{\prime \prime }\left(T\right)\approx A{\left({T-T}_{g}^{*}\right)}^{-\left(\gamma^{\prime }=1\right)}+\left[\varepsilon_{min}^{\prime \prime }+a\left({T-T}_{g}^{*}\right)\right],$$

where $A,\;a,\;\varepsilon_{min}^{\prime \prime }$ are constant amplitudes, and $T_{g}^{*}$ is the extrapolated singular temperature. 

It is visible in Figure 6 that Equation (14) offers a fair portrayal, even up to ~25 K above $T_{g}$. The form of Equation (14) is characteristic of *Critical Phenomena Physics*, for pretransitional/critical effects on approaching a continuous or weakly discontinuous phase transition [80,81,82]. They originate from pretransitional fluctuations—heterogeneities. The term in the square bracket can be considered a non-critical (molecular) background effect [64].

Results of the parameterization via Equation (14) are shown in Figure 6, with parameters given in Table 2. It also contains the $\chi^{2}$ fitting quality parameter, showing relative distortions from the fitted function. When the fitted range in Figure 6 is decreased, the values of $\chi^{2}$ do not significantly change. When the fitted range is increased above the domain indicated in Figure 6, the values of $\chi^{2}$ permanently raise. Using values given in Table 2, one can estimate the ‘discontinuity’ $\Delta T_{g}^{*}=T_{g}-T_{g}^{*}$. It is in the range of ~1.7 K for the E7 + 0.05% BaTiO_3_ nanocolloid to ~1.9 K for the E7 + 0.1% BaTiO_3_ nanocolloid.

For precritical effecst, supplementary correction-to-scaling terms are often advised when shifting away from the critical point. However, it is not the case of the mean-field-type behavior where corrections-to-scaling terms are fundamentally absent, as the consequence of their origins matched the basics of the Ginzburg criterion [64,80,81,82]. The existing evidence for various systems and properties explicitly shows that it can extend by tens of Kelvins from the phase transition without corrections-to-scaling terms [64,80,81,82]. Notably that mean-field-type behavior (MF1, MF2) manifests through pretransitional effects described by exponents that are small integer numbers or their ratios [80]. In this respect notable are theoretical model analysis suggesting the mean-field nature of the glass transition [8,9,10]. 

### 3.4. DC Electric Conductivity in the Isotropic Liquid and Supercooled Nematic

**Figure 7:**
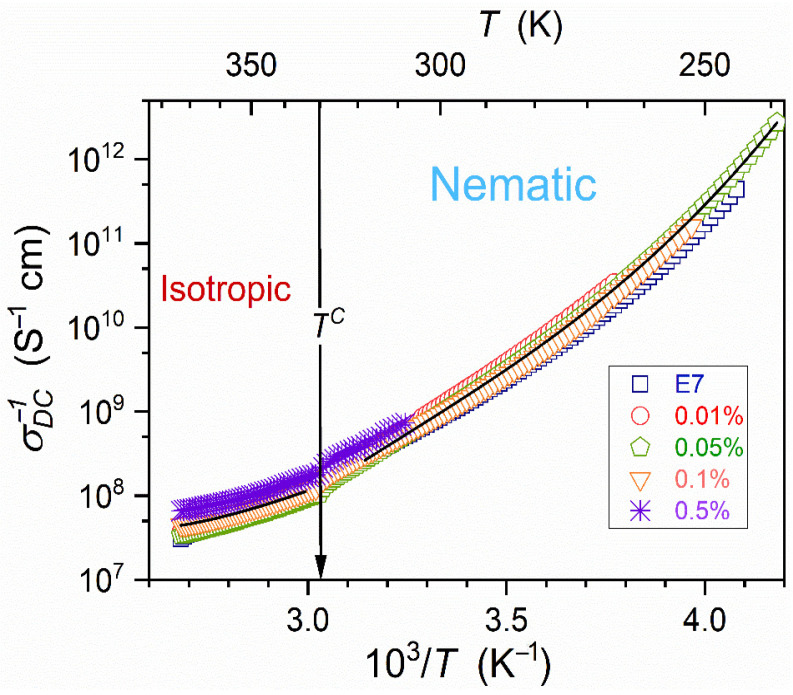
Arrhenius scale presentation of DC electric conductivity in the isotropic liquid and nematic phases of E7 and related nanocolloids. Curves are related to Equations (15) and (17).

Figure 7 presents the temperature evolution of DC electric conductivity, reflecting translational processes. The nonlinear evolution in the Arrhenius scale used in Figure 7 shows the Super/Non-Arrhenius pattern of changes. For all tested NP concentrations, related $\sigma \left(T\right)$ dependencies almost overlap and can be approximated by the conductivity-related counterpart of MYEGA Equation (13a), namely:

(15) $${\left[\sigma \left(T\right)\right]}^{-1}={\left(\sigma_{\infty }\right)}^{-1}exp\left(\frac{E_{a}^{\sigma }\left(T\right)}{RT}\right)\Rightarrow \;{\left[\sigma \left(T\right)\right]}^{-1}={\left(\sigma_{\infty }\right)}^{-1}exp\left(\frac{C}{T}exp\left(\frac{K^{\prime }}{T}\right)\right).$$

Following refs. [11,87], for vitrifying systems, one can also consider the ‘universalistic’ pattern for the steepness index, related to the apparent activation enthalpy:

(16) $$H_{a}^{\sigma }\left(T\right)=\frac{1}{R}\frac{d{ln\sigma }^{-1}\left(T\right)}{d\left(1/T\right)}=\frac{1}{R}\frac{\left[d\sigma^{-1}\left(T\right)/\sigma^{-1}\left(T\right)\right]}{d\left(1/T\right)}\approx \frac{\Phi }{T-T^{+}}\Rightarrow {\left[H_{a}^{\sigma }\left(T\right)\right]}^{-1}=\Phi^{-1}T-\left(\Phi^{-1}T^{+}\right),$$

where $\Phi ,\;T^{+}=const$ and $T^{+}$ is the singular temperature.

The above equation also shows the link to ‘relative changes’ of the considered dynamic property in the given case: $dln\sigma^{-1}\left(T\right)=d\sigma^{-1}\left(T\right)/\sigma^{-1}\left(T\right)$. 

Recalling the discussion presented in refs. [82,87], the isotropic liquid phase estimation can be related to the so-called dynamic crossover temperature, matched to a hypothetical ‘magic’ time scale: namely $T^{+}\left(isotropic\right)\approx T_{B}$. Novikov and Sokolov [88] suggested a hypothetical ‘universal’ crossover in the previtreous domain related to the time scale $\tau \left(T_{B}\right)=10^{-7\pm 1}\;s$. Nevertheless, their analysis is biased by assuming that dynamics in low-temperature and high-temperature regions of the previtreous domain are described by the VFT relation, questioned recently [11].

The behavior described by Equation (16) directly leads to the qualitatively new description of the previtreous dynamics, linking the critical-like and activated (such as the SA Equation (1)) behavior. Following ref. [87] by Drozd-Rzoska, one obtains:

(17) $$\sigma^{-1}\left(T\right)=C\times {\left(t^{-1}expt\right)}^{\Omega }=C\times {\left(\frac{T-T_{g}^{*}}{T}\right)}^{-\Omega }exp\left(\Omega \frac{T-T_{g}^{*}}{T}\right)$$

where C,Ω=const, $T>T_{g}$ and the singular temperature $T_{g}^{*}<T_{g}$.

**Figure 8:**
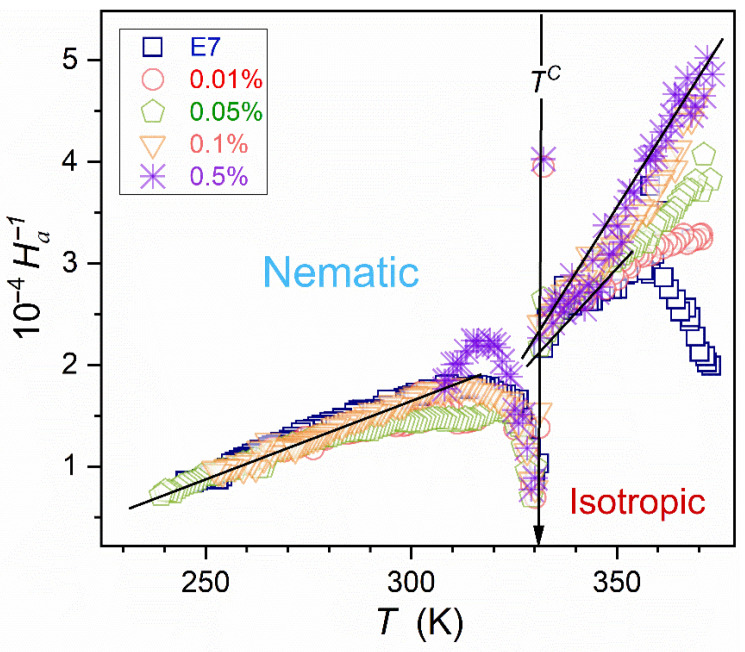
Temperature changes of the reciprocal of apparent activation enthalpy (steepness index) $H_{a}/R=dln\sigma \left(T\right)/d\left(1/T\right),$ determined using experimental data given in Figure 7. The lines show the behavior related to Equation (16).

Figure 8 validates the portrayal of the apparent activation enthalpy by Equation (16) for E7 and related nanocolloids, with the convergence when approaching $T_{g}$ and $T^{+}\approx 196\;K$. Such a description also appears in the isotropic liquid phase, with $T^{+}\approx 298\;K$ for pure E7 and $T^{+}\approx 287\;K$ for x=0.5% nanocolloid.

Following ref. [87], $T_{g}^{*}=T^{+}$; i.e., singular, critical-like temperatures in Equation (16), and easily determined by the linear regression fit viaEquation (17)-coincide. It essentially simplifies the analysis using Equation (17). The power exponent in Equation (17) can range from Ω~4 to Ω~25. For $\Omega \;\sim \;\left(4–10\right)$, the critical-like term dominates, whereas for Ω>20, the exponential (‘activated’) term is essential [87]. For the tested case of E7 rod-like (uniaxial) nematic mixture and related nanocolloids Ω≈9 in the supercooled nematic phase and Ω=3–4 in the isotropic liquid phase. Both values indicate the dominance of the first, critical-like term for dynamics described via Equation (17). For the isotropic liquid phase, such behavior correlates with the mode-coupling theory (MCT) prediction for the critical-like dynamics generally expected in the high-temperature region of glass-forming systems [7,8,9]. 

The explicit critical-like behavior in the low-temperature region of the previtreous domain, close to $T_{g}$, was predicted by Colby and Erwin [89,90,91], with the universal ’critical’ exponent ϕ=9. This value fairly agrees with the value of the exponent Ω for E7 and related nanocolloids reported above, within the limit of the experimental error.

Colby and Erwin’s ‘dynamic scaling model’ [89,90,91] was formulated as hypothetically universal for arbitrary glass-forming material. Notwithstanding, it has been fundamentally questioned following a thorough analysis of experimental data [92]. However, in the last decade, it was pointed out that the critical-like description has a clear preference over the VFT (Equation (1)) for glass-formers composed of uniaxial molecules ([11,87], and refs. therein). This is also the case of glass-forming E7 and related nanocolloids, where the uniaxiality of rod-like LC molecules is the generic feature.

## 4. Discussion: Glass Transition and Near-Critical Discontinuous Transitions

A unique result of this work is the explicit evidence for the anomalous, critical-like (Equation (14)) behavior of the loss curve maximum in the immediate vicinity of $T_{g}$.

It recalls the long-standing discussion regarding a possible relationship between the glass transition and a hypothetical hidden critical point [9,10,11,93,94,95,96,97,98,99,100,101,102,103,104,105]. The essential problem of these model discussion is the lack of the experimental evidence for the critical-type behavior near $T_{g}$. 

In *Critical Phenomena Physics*, the experimental evidence for pretransitional effects with the critical-type portrayal constitutes the essential reference and proving the driving role of pretransitional fluctuations-heterogeneities [64,80,81].

The results for $\varepsilon_{peak}^{\prime \prime }\left(T\right)$ changes presented in Figure 6 and parameterized via Equation (14), as well as the very recent communication in ref. [99], can be considered the unique evidence of the explicit ‘critical-type’ behavior in the previtreous region near $T_{g}$.

To discuss these results, it is worth recalling the case of isotropic–nematic (I-N) weakly discontinuous phase transition, which is also the topic of this report. Notable that the isotropic liquid phase above $T_{I-N}$ is also considered a specific model system for the glass transition phenomenon [100,101,102,103,104,105,106].

Let us recall basic features of the critical-like behavior in the isotropic liquid phase of nematogenic LC compounds near the weakly discontinuous I-N phase transition:

1. Dynamic properties such as the primary relaxation time τ, viscosity η, or electric conductivity σ do not show a pretransitional anomaly, but the long-range Super-Arrhenius evolution [38,39,65,66,67,68,106]. Such behavior is also visible in Figure 7, with the non-Arrhenius parameterization via Equations (16) and (17).
2. Thermodynamic properties, where heat capacity (specific heat) is the crucial example, show a well-evidenced pretransitional, critical-like ‘anomaly’ [38,39,80].
3. Static properties, such as dielectric constant, can show an explicit ‘critical’ anomaly. However, it appears only for LC systems with the permanent dipole moment approximately parallel to the long molecular axis, yielding the contrast factor CF≠0: see Figure 2 and Figure 3, and Equations (6) and (8) above and refs. [65,66,67,68,77,78,79]. For LC molecules with the perpendicular (transverse) arrangement of the dipole moment, the pretransitional effect is absent: in such a case, CF≈0 [68].
4. ‘Nonlinear’ properties, coupled to a four-point correlation function and related to such properties as NDE/NDS or the Kerr effect (KE): The pretransitional, ‘critical’ effects are always observed in the isotropic phase [23,38,39,76,82,106]. NDE, NDS, and KE are inherently associated with a strong electric field, which impacts fluctuations and their surroundings, creating a contrast factor between them; i.e., CF≠0. Definitions of these properties show that the contribution related to fluctuations is explicitly extracted.

The comparison of the behavior experimentally evidenced in the previtreous domain of glass-formers and in the isotropic phase of LC nematogens yields following conclusions:

Recalling (1)—Similar SA changes of $\tau \left(T\right)$, $\eta \left(T\right)$ or $\sigma \left(T\right)$ [4,5,6,7,8,9,10,11].
Recalling (2)—Recently, in the previtreous domain, the critical-like behavior of the configurational entropy and the associated contribution of the heat capacity has been evidenced. It was possible due to the innovative distortion-sensitive analysis [11,17].
Recalling (3)—Recently, hallmarks of dielectric constant pretransitional behavior have been noted for 8*OCB, an LC compound that can be supercooled down to $T_{g}$ in the isotropic phase [99]. Notable are extreme problems with such tests since they require $\varepsilon^{\prime }\left(T\right)$ scans for extremely low frequencies.
Recalling (4)—Generally, for experimental methods coupled to four-point correlation functions—like nonlinear dielectric spectroscopy NDS or nonlinear dielectric effect NDE—the response from ‘heterogeneities’ is registered. However, available data do not allow the consideration of temperature-related pretransitional effects ([6,9] and refs therein). It is associated with the ultraviscous/ultraslowed nature of the previtreous domain, yielding very challenging experimental requirements [6]. Nevertheless, the ‘ultraviscous limitation’ is minimized for plastic crystal-forming systems, like the Orientationally Disordered Crystals (ODICs). For NDE, the explicit critical-type effects were detected in glass & ODIC forming cyclooctane [23]. Moreover, its description can be correlated with pretransitional effect modeling for the isotropic phase of nematogens [23].

The above comparison shows notable similarities between the behavior in the isotropic liquid phase near the isotropic liquid–nematic weakly discontinuous phase transition and the previtreous domain of glass-forming systems.

In *Critical Phenomena Physics*, relatively broad supercritical and subcritical domains are shaped by collective critical fluctuations [80,81,82]. These fluctuations exhibit features of the subsequent, approaching phase. In the case of the isotropic liquid phase of LC nematogens, they are prenematic fluctuations in the isotropic liquid surrounding, i.e., symmetries related to fluctuations and their surrounding essentially differs.

Assuming that ‘dynamical heterogeneities’ observed in glass-forming systems near $T_{g}$ are related to precritical/pretransitional fluctuations, they should show features of the next, approaching phase; i.e., the amorphous solid, in the given case. Hence, in the previtreous domain, near $T_{g}$, one can consider solid amorphous fluctuations/heterogeneities submerged in the isotropic ultraviscous/ultraslowed surrounding.

For such a case, there is no symmetry difference between ‘fluctuations-heterogeneities’ and their surrounding. Only the appearance of bondings supporting solidification, within fluctuations, can be expected. It means that for the majority of standard physical monitoring methods, the contrast factor *CF* ≈ 0. It can explain the puzzling/negligible evidence of pretransitional effects in the previtreous domain. The primary relaxation time and DC electric conductivity detected in BDS studies are related to the average response from individual molecules, whose ability to orientation or translation can be only slightly ‘distorted’ by the ‘frustration’ caused by fluctuations/heterogeneities appearing or disappearing. Such weak frustration can explain the Super-Arrhenius and non-Debye dynamics in glass-forming systems. A similar impact of the ‘fluctuating surrounding’ on single molecule-related dynamics occurs in the isotropic phase of LC nematogens [64,65,66,67,68,106].

One can expect the appearance of the $\varepsilon_{peak}^{\prime \prime }\left(T\right)$ pretransitional effect, can be possible if the response regarding energy requirements for the reorientation process is different for fluctuations/heterogeneities and their surroundings, thus yielding CF≠0. Such impact factor can be expected for anisotropic molecules.

In supercooled r E7 (rod-like: inherently anisotropic), the solidification/vitrification is related to the gradual freezing of the translational disorder for the locally orientationally ordered nematic phase. It means that previtreous/pretransitional fluctuations with a ‘frozen’ prenematic arrangement are ‘supported’ by intermolecular bondings. It can yield a generic contrast factor CF≠0 and then previtreous anomaly of for $\varepsilon_{peak}^{\prime \prime }\left(T\right)$ in E7 based systems. For E7-based nanocolloids, the addition of nanoparticles can influence the ordering/arrangement and bonding process, changing the values of $\varepsilon_{peak}^{\prime \prime }\left(T\right)$, as shown in Figure 6.

Such a mechanism could not be effective for non-anisotropic, near-globular molecular systems, like glycerol—the standard glass-former, and for the vast majority of molecular glass-formers. 

## 5. Conclusions

This report aims to fill the research gap for the previtreous behavior in LC-based nanocolloids. BDS studies were carried out in the E7 glass-forming LC mixture composed of rod-like molecules and in E7 nanocolloids with BaTiO_3_ nanoparticles dispersion. First, pretransitional changes of dielectric constant in the isotropic liquid phase have been presented. They follow the pattern known for nematogenic compounds with the permanent dipole moment approximately parallel to the long molecular axis for E7 and related nanocolloids. This pretransitional effect is related to prenematic fluctuations due to the weakly discontinuous character of the I-N transition.

Before the studies presented above, only a few reports related to E7-based nanocolloids were reported [43,49,71,106,107,108,109,110,111,112,113,114,115]. However, they do not cover the temperature range discussed in the report or attempt to detect pretransitional effects. Regarding dynamics, only the description via the ‘effective’ VFT portrayal was considered.

The dominant part of studies on the previtreous behavior in glass-forming systems is focused on dynamics, tested via the primary relaxation time and DC electric conductivity using broadband dielectric spectroscopy.

For E7 and related nanocolloids, the fair portrayal of $\tau \left(T\right)$ and $\sigma \left(T\right)$ changes via the Super-Arrhenius MYEGA equation have been shown (Equations (13) and (15)). The universalistic pattern of changes for the apparent activation enthalpy is notable. It coincides with the Arrhenius scale presentation’s steepness index (fragility). This property can be considered the metric of $\tau \left(T\right)$- or $\sigma \left(T\right)$-relative changes in the previtreous domain (Equation (16)). Notably, these properties are associated with orientational and translational dynamics, respectively [7,11]. The primary relaxation time is related to the time scale associated with the primary loss curve peak in the $\varepsilon^{\prime \prime }\left(f\right)$ spectrum [7,11]. The second coordinate of the peak is the loss curve maximum $\varepsilon_{peak}^{\prime \prime }\left(T\right)$. It reflects the energy required for the orientation [66,67]. Surprisingly, the evolution of $\varepsilon_{peak}^{\prime \prime }\left(T\right)$ is hardly, if at all, evidenced in studies on previtreous dynamics. This report shows that $\tau \left(T\right)$ changes in supercooling E7-based nanocolloids, for different concentrations of nanoparticles, overlap. However, previtreous changes of $\varepsilon_{peak}^{\prime \prime }\left(T\right)$ strongly depend on the concentration of nanoparticles. Moreover, for this property, the unique critical-like effect near $T_{g}$, extending up to $\sim T_{g}+20\;K$, appears. It is shown in Figure 6, with the parameterization via Equation (14).

The discussion presented in the given report indicates significant similarities for pretransitional effects in the isotropic liquid phase, near the weakly discontinuous I-N phase transition, and in the previtreous domain of glass-forming systems near the glass transitions. For the latter, the uniaxiality of molecules in the glass-forming system can support the detection of pretransitional effects. It is notable that I-N transition is associated with the symmetry change, whereas for the glass transition, a unique case of the ‘amorphous’ transition avoiding a symmetry change may be expected. Such specification can also directly explain the ‘diffused’ temperature characterization in the surrounding of $T_{g}$ for some physical properties.

The results discussed in this report can indicate a possible milestone significance for studying previtreous phenomena in glass-formers composed of anisotropic molecules, where the addition of nanoparticles offers a supplementary possibility of testing the impact of local nano-frustrations.

## Figures and Tables

**Table 1:** Values of parameters obtained for temperature changes of $d\varepsilon \left(T\right)/dT$ are parameterized by Equation (8). Results of fitting are shown graphically in Figure 3. Constant value α=φ−1=1/2 was assumed to facilitate nonlinear fitting with minimal errors. The latter is related to the last significant number for each presented value.

E7+x% NPs	*a*	B	$T^{*}(K)$	φ−1
x=0%	−0.039	0.13	329.5	1/2
x=0.01% , x=0.05%	−0.054	0.18	327	1/2
x=0.1%	−0.10	0.46	319	1/2
x=0.5%	−0.115	0.54	316	1/2

**Table 2:** Values of parameters related to Equation (14) and Figure 6 for the pretransitional anomaly near $T_{g}=211.2\;K$, estimated via the condition $\tau \left(T_{g}\right)=100\;s$. The table contains $\chi^{2}$ parameter showing the relative fitting error, approximately constant in the fitting temperature range shown in Figure 6. The increase in this range raises values of $\chi^{2}$. The data in the table are obtained assuming for the exponent γ=1, which allowed minimizing errors of remaining parameters. The ‘free fits’ are related to the error ±0.03 for the exponent γ.

E7 + NPs (x%)	$10^{7}\times A\left(K\right)$	$\varepsilon_{min}^{\prime \prime }$	$a\left(K^{-1}\right)$	$T_{g}^{*}$ (K)	Exponent γ′	$\chi^{2}$
x=0%	8.54	0.46_2_	0.72_0_	209.4	1±0.03	0.989
x=0.01%	8.38	0.45_4_	0.71_7_	209.3		0.9882
x=0.05%	8.20	0.44_0_	0.71_4_	209.5		0.983
x=0.1%	7.50	0.40_7_	0.71_0_	209.3		0.991

**Figure A1:**
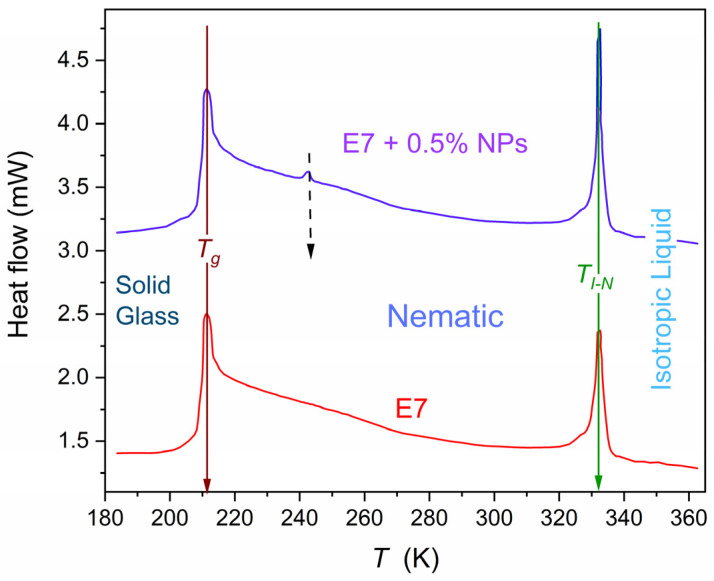
DSC scans for nematogenic E7 mixtures and related nanocolloids with BaTiO_3_ nanoparticles. The clearing temperature (I-N), discontinuous phase transition, and glass temperatures are indicated. In the nanocolloid, the dashed arrow indicates a phase transition within the supercooled nematic phase. For the latter, note the evidence in Figure 2.

## Data Availability

All data are available directly from the authors following reasonable request. They are also deposited in the public open-access REPOD database (Poland).

## Appendix A

### Appendix A.1. The Fragility Concept in Glass-Forming Systems

Almost 4 decades ago, Austin Angell [107] proposed the plot enabling a common presentation of anomalous viscosity $\eta \left(T\right)$ changes in supercooled liquids and polymers on approaching glass transition. Soon later, he extended it for the primary relaxation time $\tau \left(T\right)$ determined from broadband dielectric spectroscopy studies [108]. It was related to ${log}_{10}\eta \left(T\right)$ or ${log}_{10}\tau \left(T\right)$ vs. $T_{g}/T$, plots supplemented by the assumption $\eta \left(T_{g}\right)=10^{13}\;\mathrm{Poise}$ and $\tau \left(T_{g}\right)=10^{13}\;\mathrm{Poise}$. The latter causes that $\eta \left(T\right)$ and $\tau \left(T\right)$ previtreous effect intersects, and the terminal point $\left[\eta \left(T_{g}\right),\;\left(T_{g}/T\right)=1\right]$ or $\left[\tau \left(T_{g}\right),\;\left(T_{g}/T\right)=1\right]$. Using numerous available empirical data, Angell showed that for the $\left(T_{g}/T\right)\to 0$ intersection of normalized previtreous changes at the ‘onset’ $\eta \left(T_{on}\right)\sim 0.1cPoise$ or $\tau \left(T_{on}\right)\sim 10^{-14}\;s$ occur, at least for low molecular weight liquids and polymers [107,108]. For such a normalized presentation, the standard Arrhenius dependence, with the constant activation energy in Equation (1) appears as a straight line extending between the boundary points marked above. For glass-forming systems, these are ‘anomalous’ changes, i.e., a series of curves located below the Arrhenius straight line, constituting a reference for the characteristic of temperature changes in the previtreous domain. For the classification of described previtreous changes, Angell introduced the metric: the slope of ${log}_{10}\eta \left(T\right)$ or ${log}_{10}\tau \left(T\right)$ dependencies at $T=T_{g}$, and named it ‘fragility’ [107,108]:

(A1) $$m=\frac{d{log}_{10}}{dT_{g}/T}$$

For the reference (‘normal’) Arrhenius behavior, m=μ=16, which is the minimal fragility related to the timescale of 16 decades and timescale change; namely, ${log}_{10}\tau \left(T_{g}\right)-{log}_{10}\tau \left(T_{on}\right)$. For very ‘fragile’ glass-formers, values m>200 take place.

The above fragility definition applied for the VFT Equation (1) leads to the following dependences [11]:

(A2) $$m=\frac{D_{T}T_{0}T_{g}}{{\left(\Delta T_{g}^{*}\right)}^{2}ln10}$$

or alternatively,

(A3) $$m=\mu \left(1+\frac{ln10}{D_{T}}\right)\Rightarrow D_{T}=\frac{\mu ln10}{m-\mu }$$

where $\Delta T_{g}^{*}=T_{g}-T_{0}$, $\mu =m_{min}={log}_{10}\tau \left(T_{g}\right)-{log}_{10}\tau_{\infty }$; assuming $\tau_{\infty }=10^{-14}\;s$ for the prefactor in the VFT relation, one obtains μ=16; $T_{g}$ means the glass temperature, $T_{0}$ is the VFT singular temperature, and then $\Delta T_{g}^{*}$ can be considered as an apparent discontinuity of the glass temperatures [11].

The most commonly used right-hand part of Equation (A3) was first shown in ref. [108] as $m=16+590/D_{T}$ [108]. Hence, the fragility strength coefficient in the VFT Equation (1) can be considered as the alternative metric for the basic fragility parameter. Notably, the Angell plot has become the hallmark of *Glass Transition Physics*, reinforcing the belief in glass transition’s unique ‘dynamic’ nature [2,3,4,5,6,7,8,9,10,11]. The importance of the fragility parameter has become one of the central areas of research in this field [2,3,4,5,6,7,8,9,10,11,109,110,111,112]. The first consistent equivalents of the Angell plot and fragility for pressure-related previtreous effects were proposed by the authors of the given report (A. Drozd-Rzoska and S.J. Rzoska) in ref. [113].

### Appendix A.2. Supplementary DSC Studies

The plot below shows the result of DSC scans for the pure nematogenic LC mixture E7 and for the nanocolloid E7+0.5% BaTiO_3_ nanoparticles (paraelectric, d=50 nm).

Tests were carried out using a home-made DSC facility using 0.5 g of the sample. The temperature control was supported by the Quattro Novocontrol unit, the same as for BDS studies in the given report. Copper-constant thermocouples supported temperature measurements. The results are for 10/min scan, but 10× slowing down of the scan did not change the pattern. For pure E7, note the agreement with the results presented in refs. [114,115]. Note the indication for a discontinuous transition within the nematic phase in the nanocolloid, which is also evidenced by the dielectric constant scans presented above. It can suggest a change in the arrangement of permanent dipole moments coupled to rod-like molecules.
